# Unbiased descriptor and parameter selection confirms the potential of proteochemometric modelling

**DOI:** 10.1186/1471-2105-6-50

**Published:** 2005-03-10

**Authors:** Eva Freyhult, Peteris Prusis, Maris Lapinsh, Jarl ES Wikberg, Vincent Moulton, Mats G Gustafsson

**Affiliations:** 1 The Linnaeus Centre for Bioinformatics, Uppsala University, Box 598, S-751 24 Uppsala, Sweden; 2 Department of Pharmaceutical Biosciences, Uppsala University, Box 591, S-751 24 Uppsala, Sweden; 3 Department of Engineering Sciences, Uppsala University, Box 528, S-751 20 Uppsala, Sweden

## Abstract

**Background:**

Proteochemometrics is a new methodology that allows prediction of protein function directly from real interaction measurement data without the need of 3D structure information. Several reported proteochemometric models of ligand-receptor interactions have already yielded significant insights into various forms of bio-molecular interactions. The proteochemometric models are multivariate regression models that predict binding affinity for a particular combination of features of the ligand and protein. Although proteochemometric models have already offered interesting results in various studies, no detailed statistical evaluation of their average predictive power has been performed. In particular, variable subset selection performed to date has always relied on using all available examples, a situation also encountered in microarray gene expression data analysis.

**Results:**

A methodology for an unbiased evaluation of the predictive power of proteochemometric models was implemented and results from applying it to two of the largest proteochemometric data sets yet reported are presented. A double cross-validation loop procedure is used to estimate the expected performance of a given design method. The unbiased performance estimates (*P*^2^) obtained for the data sets that we consider confirm that properly designed single proteochemometric models have useful predictive power, but that a standard design based on cross validation may yield models with quite limited performance. The results also show that different commercial software packages employed for the design of proteochemometric models may yield very different and therefore misleading performance estimates. In addition, the differences in the models obtained in the double CV loop indicate that detailed chemical interpretation of a single proteochemometric model is uncertain when data sets are small.

**Conclusion:**

The double CV loop employed offer unbiased performance estimates about a given proteochemometric modelling procedure, making it possible to identify cases where the proteochemometric design does not result in useful predictive models. Chemical interpretations of single proteochemometric models are uncertain and should instead be based on all the models selected in the double CV loop employed here.

## Background

Current computational methods for prediction of protein function rely to a large extent on predictions based on the amino acid sequence similarity with proteins having known functions. The accuracy of such predictions depends on how much information about function is embedded in the sequence similarity and on how well the computational methods are able to extract that information. Other computational methods for prediction of protein function include structural similarity comparisons and molecular dynamics simulations (e.g. molecular docking). Although these latter methods are powerful and may in general offer important 3D mechanistic explanations of interaction and function, they require access to protein 3D structure. Computational determination of a 3D structure is well known to be resource demanding, error prone, and generally requires prior knowledge, such as the 3D structure of a homologous protein. This bottleneck makes it important to develop new methods for prediction of protein function when a 3D model is not available.

Recently a new bioinformatic approach to prediction of protein function called *proteochemometrics *was introduced that has several useful features [1-4]. In proteochemometrics the physico-chemical properties of the interacting molecules are used to characterize protein interaction and classify the proteins into different categories using multivariate statistical techniques. One major strength of proteochemometrics is that the results are obtained directly from real interaction measurement data and do not require access to any 3D protein structure model to provide quite specific information about interaction.

Proteochemometrics has its roots in chemometrics, the subfield of chemistry associated with statistical planning, modelling and analysis of chemical experiments [5]. In particular it is closely related to quantitative-structure activity relationship (QSAR) modelling, a branch of chemometrics used in computer based drug discovery. Modern computer based drug discovery is based on modelling interactions between small drug candidates (ligands) and proteins. The standard approach is to predict the affinity of a ligand by means of numerical calculations from first principles using molecular dynamics or quantum mechanics. QSAR modelling is an alternative approach where experimental observations are used to design a multivariate regression model.

With *x*_*i *_denoting descriptor *i *among *D *different descriptors and *y *denoting the biological activity, (linear) QSAR modelling aims at a linear multivariate model

*y *= **w**^*T *^**x **= *w*_0 _+ *w*_1_*x*_1 _+ *w*_2_*x*_2 _+ ... + *w*_*D*_*x*_*D *_    (1)

where **w **= [*w*_0_, *w*_1_, *w*_2_,..., *w*_*D*_]^*T *^are the regression coefficients and **x **= [1, *x*_1_, *x*_2_,..., *x*_*D*_]^*T*^. The activity *y*, may be the binding affinity to a receptor but may also be any biological activity e.g., the growth inhibition of cancer cells. In comparison with numerical calculations from first principles and similar approaches, the main advantages of QSAR modelling are that it does not require access to the molecular details of the biological subsystem of interest and that information can be obtained directly from relatively cheap measurements.

The joint perturbation of both the ligand and protein in proteochemometrics yields additional information about the different combinations of ligand and protein properties for an interaction than can be obtained in conventional QSAR modelling where only the ligand is perturbed. In recent years, various other bioinformatic modifications of conventional QSAR modelling have been reported. These include simultaneous modifications of the ligand and the chemical environment (buffer composition and/or temperature) in which the interaction take place [6-8], and three-dimensional QSAR modelling of protein-protein interactions that directly yields valuable stereo-chemical information [9].

Although proteochemometrics has already proven to be an useful methodology for improved understanding of bio-chemical interactions directly from measurement data, the quantitative proteochemometric models designed so far have not yet been subject to a detailed and unbiased statistical evaluation.

A key issue in this evaluation is the problem of overfitting. Since the number of ligand and protein properties available is usually very large, to avoid overfitting, one has to constrain the fitting of the regression coefficients. For example, in ridge regression [10], a penalty parameter is tuned based on data to avoid overfitting, and in partial least squares (PLS) regression [11-13] the overfitting is controlled by tuning the number of latent variables employed. In proteochemometrics as well as in many QSAR studies reported, the performance estimates reported are obtained as follows: 1) Perform a *K*-fold CV for different regression parameters, 2) Select the parameter value that yields the largest estimated performance value, and 3) Report the most promising model found and the associated performance estimate. Although this procedure may seem intuitive and may yield predictive models (as we in fact demonstrate below) the performance estimates obtained in this way may be heavily biased. Interestingly, this problem was recently addressed in the context of conventional QSAR modelling [14], and has also been discussed in earlier work, see [15,16].

As an alternative or complement to constraining the regression coefficients, one may also reduce the variance by means of variable subset selection (VSS). In QSAR modelling, many algorithms for VSS have been proposed based on various methodologies, for example optimal experimental design [17,18], sequential refinements [19], and global optimization [20]. VSS is used to exclude variables that are not important for the response variable, in the process of model building. Variables that are not important receive low weights in both a PLS and a ridge regression model, however if the fraction of unimportant variables is very large [21] the overall predictive power of the model is reduced. In this case VSS can improve the predictivity. However, if the fraction of unimportant variables is rather small, the quality of the model will not be improved by using VSS, it might on the contrary be slightly reduced. However, the interpretability of the model will in both cases be improved.

Although many of the advanced algorithms for VSS are powerful, they are all computationally demanding. Therefore, in order to keep the computing time down in our use of the double loop cross validation procedure employed here, conceptually and computationally simple algorithms for VSS were used instead of the more advanced ones presented, e.g. in [17-20]. Most likely, the more advanced algorithms would yield more reliable models with even higher predictive power than for the models designed here. However, the main issue of interest in this paper is to confirm the potential of proteochemometrics.

In previous reported proteochemometrics modelling, all available examples were used in the VSS. These were split into *K *separate parts and a conventional *K*-fold cross validation (CV) was performed. However, since all available examples were used, there were no longer any completely independent test examples available for model evaluation. Interestingly, this problem of introducing an optimistic selection bias via VSS was recently also pointed out in the supervised classification of gene expression microarray data [22].

**Figure 1:**
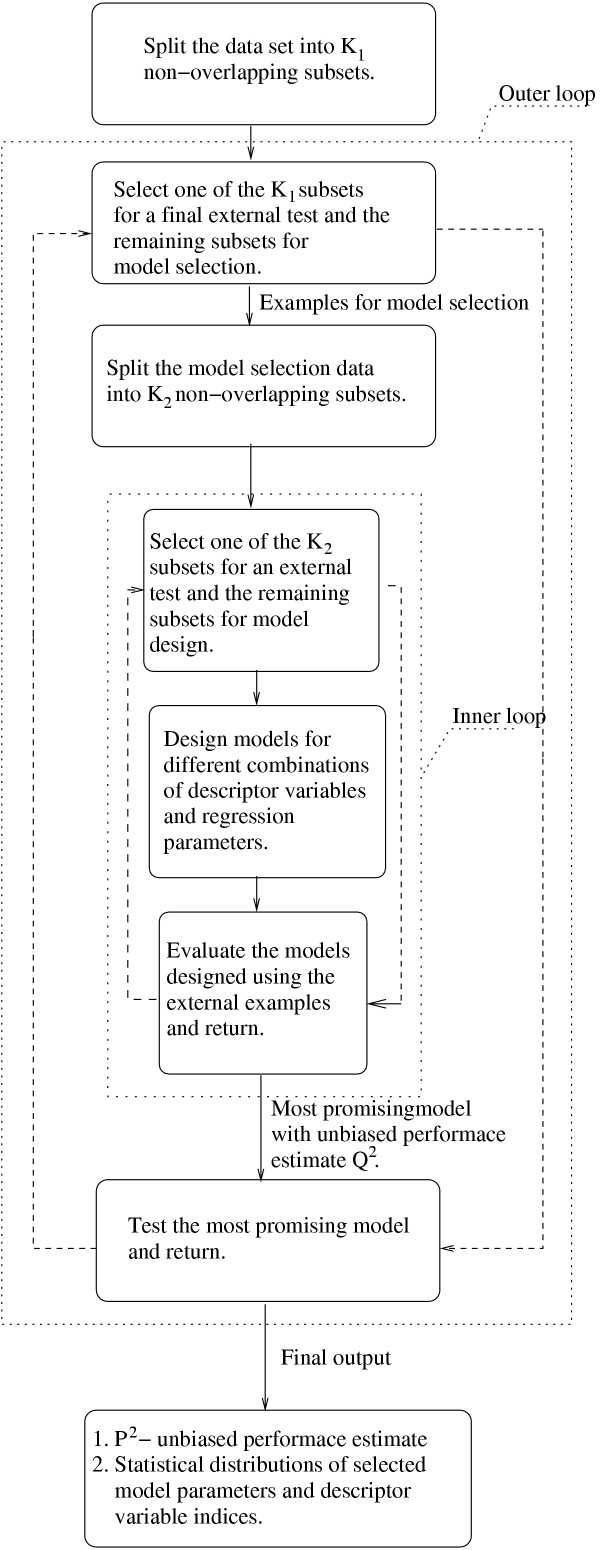
**Overview of double loop. **An overview of the double loop CV procedure used to obtain the desired unbiased performance estimate *P*^2^. In the embedded inner CV loop, the most promising model is selected which yields the largest unbiased performance estimate, *Q*^2^. In the outer CV loop, external test examples are kept outside the inner loop and are only used to test the most promising model found in the inner loop. Note that the estimate *P*^2 ^reflects the average performance of the modelling procedure employed in the inner loop and that the estimate is based on many different models designed in the inner loop.

In this paper we employ a procedure that can be used to perform unbiased statistical evaluations of proteochemometric and other QSAR modelling approaches. An overview of this so-called double loop CV procedure is presented in Figure 1, and may be regarded as a refinement of the current practice in proteochemometrics in the following respects:

1. *K*_1 _different variable subset selections are performed, one for each step in the outer CV loop. This avoids optimistic selection bias.

2. The best performance estimates (*Q*^2^) found in the inner loop by means of *K*_2_-fold CV are computed, but not reported as the model's performance estimate. This avoids the second optimistic selection bias mentioned above.

3. An unbiased performance estimate, *P*^2^, is computed in the outer loop and is reported as the performance estimate of the modelling approach defined by the procedure in the inner loop (the methods of VSS, regression, and model selection employed). *P*^2 ^is the result of different models that are designed and selected in the inner loop. It reflects the performance that one should expect on average.

4. Repeated *K*_1_-fold CVs which yield information about the robustness in the results obtained (presented as confidence intervals).

In addition to these refinements, this work also demonstrates the potential of fast and straight forward alternative methods for VSS and regression in the inner loop. Moreover, it indicates that the performance estimates reported by certain software packages for QSAR may be quite misleading.

We reanalyzed two of the largest proteochemometric data sets yet reported. The first data set is presented in [2] and contains information about the interactions between 332 combinations of 23 different compounds with 21 different human and rat amine G-protein coupled receptors. In total, there are 23 × 21 = 483 possible interactions and the basic task is to fill in the 483-332 = 151 missing values. The second data is presented in [23] and contains information about the binding of 12 different compounds (4-piperidyl oxazole antagonists) to 18 human α_1_-adrenoreceptor variants (wild-types, chimeric, and point mutated). As for the first data set, there are not interaction data available for all the 12 × 18 = 216 possible interactions, but for 131, see [24] for more details about this data set. Below these two data sets are referred to as the *amine data set *and the *alpha data set*, respectively.

## Results

### Software

Computer programs were written in MATLAB (Mathworks Inc., USA) to integrate the double loop procedure in Figure 1 with robust multivariate linear regression using partial least squares (PLS) regression and ridge regression. These programs also contained two simple and fast methods for variable ranking called corrfilter and PLSfilter. For details, see the Methods section.

### Parameters

The joint variable selection and PLS tuning performed in the inner *K*_2_-fold CV loop was performed with *K*_2 _= 5. The different values of *N*_*D *_(the number of molecular descriptors) evaluated were 10, 20, 50, 100, 200, ..., 1000, 1500, 2000, ..., 6000 for the amine dataset and 10, 20, 50, 100, 200, ..., 1000, 1500, 2000 for the alpha data set. The values of *N*_*L *_considered were either the number of latent variables 1, 2, ..., 8, for both the amine and alpha data set or the degree of RR penalty 0, 0.5, 1.0, ..., 3.0 for the amine data set and 10, 50, 100, 150, 200 for the alpha data set. In the outer *K*_1_-fold CV loop, the same number of splits (*K*_1 _= 5) was used as in the inner loop. On the global level, the complete experiments were performed 100 times using different random partitions of the complete data sets.

### Unbiased predictive power

**Figure 2:**
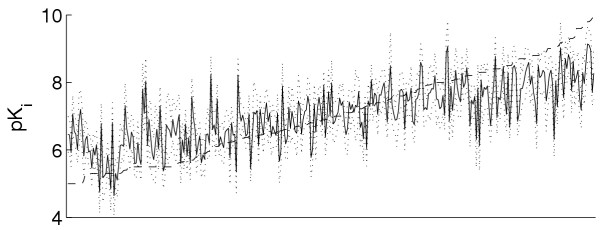
**External predictions for amine data set. **External predictions for amine data set sorted according to growing values of *pK*_*i*_. The figure shows the experimental *pK*_*i *_values (dashed line) and the mean value of the predicted *pK*_*i *_values (solid line) with a 95 % confidence interval (dotted lines). The predictions shown in the figure are from the PLS modelling after variable selection using PLSfilter. The results indicate that the high and low *pK*_*i *_values are hard to predict.

In Table 1 a summary of the results from 100 randomly selected partitions of the complete amine data set are presented in the form of the mean values and standard deviations obtained. The number of molecular descriptors and latent variables selected in the inner loop are summarized in Table 2. The average values of the biased *Q*^2 ^obtained in the inner loops look quite promising for the PLSfilter method (*Q*^2 ^= 0.90 for both PLS and RR) and is even higher than the value reported in earlier studies [2]. However, the corresponding unbiased performance estimate *P*^2 ^is much smaller (*P*^2 ^= 0.52 or 0.51 for PLS and RR, respectively). The *Q*^2 ^values for the models obtained after variable selection using corrfilter are significantly lower than when using PLSfilter, but the *P*^2 ^values are almost on the same level for the two variable selection methods when no variable selection at all is used. corrfilter reduces the number of descriptors to about one third of the initial number, but corrfilter still selects more than twice as many descriptors than PLSfilter (see Table 2). Since the main reason for variable selection is improving the interpretation of the model by reducing the number of descriptors, this indicates that one should select PLSfilter instead of corrfilter. In Figure 2, the external (unbiased) predictions used to compute *P*^2 ^for the PLS model using PLSfilter show that there is useful predictive power, but only for examples with mid-range *pK*_*i *_values. The model has poor predictability for both low and high *pK*_*i *_values, indicating that the standard design procedure used in the inner CV loop does not always yield reliable models. This confirms earlier findings [14], that maximization of the unbiased performance estimate *Q*^2 ^is not always reliable, and also indicates that unreliable designs can be detected by means of the outer CV loop employed in this work.

**Figure 3:**
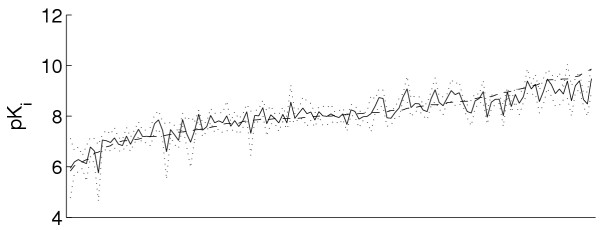
**External prediction for alpha data set. **External predictions for alpha data set sorted according to growing values of *pK*_*i*_. The figure shows the experimental *pK*_*i *_values (dashed line) and the mean value of the predicted *pK*_*i *_values (solid line) with a 95 % confidence interval (dotted lines). The predictions shown in the figure are from the PLS modelling after variable selection using PLSfilter.

The estimated performances of the models for the alpha data sets are presented in Table 3. Here both the *Q*^2 ^and *P*^2 ^values are high and the difference between the two measures is smaller than for the amine data set. This indicates a lower level of overfitting. The number of descriptors selected in the variable selection is much lower for the alpha data set (see Table 4) than for the amine data set. Both the high *P*^2 ^values and the display of the external prediction in Figure 3 show that the models have high predictive power. Also, the predictive power is significantly higher after variable selection than without. This is an example when variable selection does not only improve the model interpretability, but also the the model predictivity. The above results indicate, for example, that a combination of PLSfilter, PLS regression and model selection by maximization of *Q*^2 ^produces individual models with predictive power. The relative standard deviation of the predictive power is less than 5% for the two data sets considered. However, the number of variables selected has a relative standard deviation of 455/1933 = 25% and 69/199 = 35%, respectively. Moreover, the standard deviation in the number of latent variables (an implicit constraint on the regression coefficients) is approximately one (1.4 and 0.8) or 15%. In conclusion, the individual models are quite different but essentially all of them yield useful predictions.

### Comparisons to other programs

**Figure 4:**
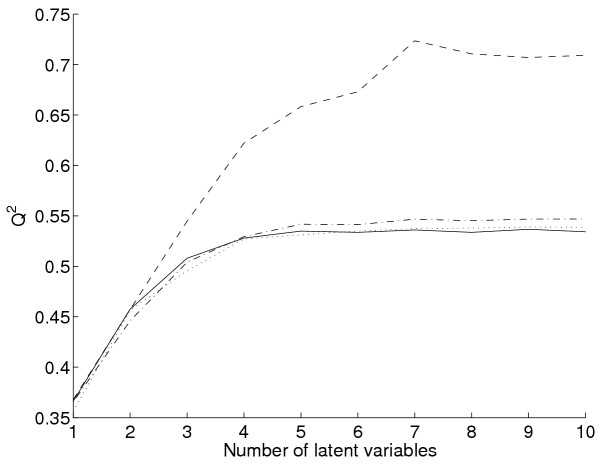
**Comparison of software. ***Q*^2 ^values obtained using different software for the prediction of affinities based on PLS models, without variable selection, for the amine data set. Between one and ten latent variables were used and SIMCA (dashed line), UNSCRAMBLER (dash-dotted line), GOLPE (dotted line) and MATLAB (solid line) were used to both build the models and evaluate them by computing *Q*^2 ^values. The SIMCA *Q*^2 ^values are much higher than the other *Q*^2 ^values.

To verify that our computations using MATLAB are comparable to computations by other programs, such as SIMCA, GOLPE and UNSCRAMBLER, models without variable selection were performed with all four approaches. In the comparison we have compared *Q*^2 ^values for models based on all descriptors built with PLS using between one and ten latent variables for the amine data set. All the *Q*^2 ^values were computed using the leave out CV method with five random groups and are presented in Figure 4.

Remarkably, the *Q*^2 ^values obtained with SIMCA 7.0 are much higher than for the other methods. This is due to the fact that SIMCA does not use the standard formula (Eq. 3) to compute *Q*^2 ^(personal communication with Umetrics), for some general information see [25].

### Robustness and interpretability

**Figure 5:**
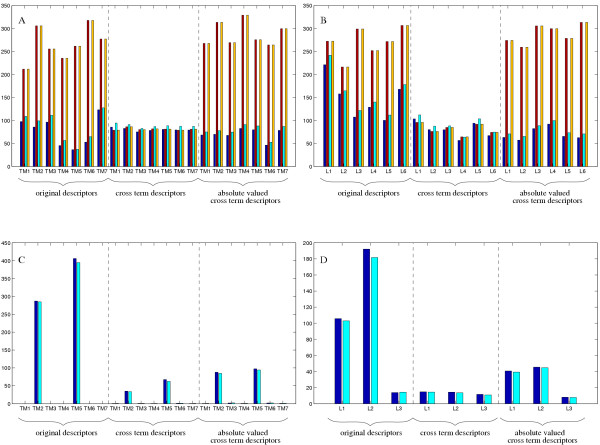
**Hit rates. A **The hit rates for the receptor blocks in the amine data set. The figure shows for each transmembrane region the hit rates for the original receptor descriptors, the cross term descriptors and the absolute valued cross term descriptors involving that transmembrane region. **B **The hit rates for the ligand blocks in the amine data set. The figure shows for each ligand descriptor block the hit rates for the original receptor descriptors, the cross term descriptors and the absolute valued cross term descriptors involving that ligand descriptor block. **C **and **D **The corresponding hit rates for the alpha data set. The blue bars show the hit rates computed for the PLS models using PLSfilter, the cyan bars show the hit rates computed for the PLS models using corrfilter, the red bars show the hit rates computed for the RR models using PLSfilter, and the yellow bars show the hit rates computed for the RR models using corrfilter

To study the robustness and interpretability of the set of models obtained using the two data sets considered, two different levels of information were computed and presented. The first level of information consists of two histograms displaying, for each ligand block (L1–L6 for the amine data set, and L1–L3 for the alpha data set (for the alpha data set the three ligand blocks correspond to the three positions of modification in the ligand)), and for each transmembrane region (TM1–TM7), how often different kinds of descriptors are selected. The histograms are based on the 500 observations obtained in the 5-fold cross validation performed 100 times using different, randomly selected, partitions of the data set. The descriptors are divided into receptor descriptors and ligand descriptors that are further subdivided into original descriptors, cross term descriptors, and absolute valued cross terms. In Figure 5, hit rates for receptor and ligand blocks in the 100 different 5-fold cross validations performed are presented, both for the original, the cross term, and the absolute valued cross term descriptors. In Figure 5 A and 5B, the results for the amine data set are presented. Figure 5 C and 5D displays the corresponding results for the alpha data set.

**Figure 6:**
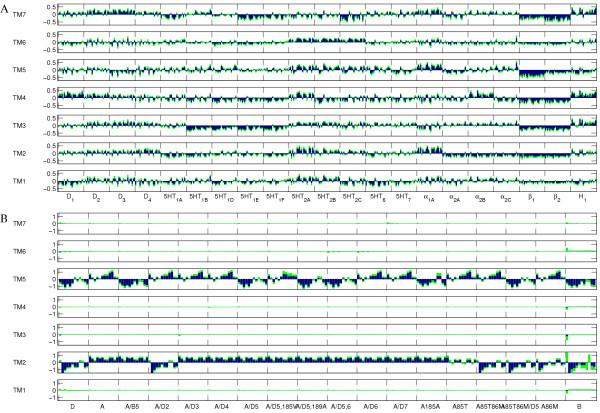
**Detailed contributions to affinity. A **Contributions of TM regions in amine GPCRs to the ligand affinity according to the proteochemometrics models created using PLS in combination with the variable selection method PLSfilter. The contributions are shown for all the 21 receptors, for each receptor 23 bars corresponding to the 23 ligands are shown (in alphabetical order i.e., Amperozide, Clozapine, Fluparoxan, Fluspirilene, GGR218231, Haloperidol, L741626, MDL100,907, ORG5222, Ocaperidone, Olanzapine, Pipamperone, Raclopride, Risperidone, S16924, S18327, S33084, Seroquel, Sertindole, Tiospirone, Yohimbine, Ziprasidone, Zotepine). The blue bars show the average contribution and the height of the green bars show one standard deviation. The average value and standard deviation were computed using all the 500 models designed (100 repeats and five blocks for each repeat). **B **Contributions of TM regions in α_1_-adrenoreceptors to the ligand affinity according to the proteochemometrics models created using RR in combination with the variable selection method PLSfilter. The contributions are shown for all the 18 receptors, for each receptor 12 bars corresponding to the 12 ligands are shown (in numerical order 1–12). The blue bars show the average contribution and the height of the green bars show one standard deviation. The average value and standard deviation were computed using all the 500 models (100 repeats and five blocks for each repeat).

The second level of information displays the average and the standard deviation of the contribution of the different TM regions in the receptors for creation of receptor-ligand affinity according to the 500 different models designed. The contributions were calculated exactly as described in [2] for a single model, and then the average and standard deviation were calculated. Therefore, the results presented in Figure 5 corresponds to Fig. 3  in [2] where the results for a single proteochemometric model were presented. As before, for each TM region, the contributions to the affinity by different ligands is displayed, this time the variance (uncertainty) information is added. The top of Figure 6 shows the detailed contributions of TM regions to affinity, for each possible combination of ligand and receptor, according to the 500 different proteochemometric models designed using the amine data set, PLS regression and the PLSfilter VSS algorithm. The bottom part displays the corresponding results for the alpha data set when employing ridge regression and the PLSfilter VSS algorithm.

## Discussion

In summary, the results reported here confirm earlier reports on the potential of proteochemometrics modelling for prediction of biological activity. It is interesting to note that the VSS did increase the predictivity of the models for the alpha data set, but not for the amine data set. The VSS for the alpha data set did also reduce the number of variables to approximately 4% of the original variables, while for the amine data set 15–38% of the variables remained after VSS. This indicates that for models where many variables receive low weights (as for the alpha data set) the VSS can significantly improve the model, whereas for a data set like the amine data set, with less low weighted variables, the VSS does not improve the model even though it can improve the interpret ability of the model.

The basic goal of proteochemometric modelling is to obtain a single quantitative model that can predict biological activities accurately and which can be easily interpreted biochemically. In this context, it is important to stress that the only role of the outer loop employed in this work is to obtain unbiased estimates of the average performance of the design procedure considered in the inner loop. The additional random splitting of the data sets is used on top of this to gain information about the stability in the performance estimates. Thus, for procedures in the inner loop that yield small variances around a high average of *P*^2^, there is statistical support that a single design will yield useful predictions. In order for a single model to be chemically interpretable as well, all the models selected in the inner loop should yield approximately the same number (same set) of variables and the constraints on the regression coefficients (e.g., the number of latent variable in PLS regression) in all models should be approximately equal. With this in mind, the results presented in this work indicate that it is possible to design single proteochemometric models with predictive power based on the two data sets considered but that there is a relatively large variance (from one design set to another) in the variables selected and the constraints put on the regression coefficients. This indicates that although a single proteochemometric model would be useful for predictions, a detailed chemical analysis of such a model would be uncertain. More reliable information should be gained from a careful joint analysis of all the models (and their variables) selected in the inner loops of the different evaluations performed. For example, as briefly discussed in [9], the variables selected with the highest frequency should be of great interest. Thus, systematic and simultaneous biochemical analyses of all the models selected in the inner loops of this kind are required. For illustrative purposes of the complexity and potential of such analyses, here we have presented frequency distributions indicating which variable blocks are selected frequently in the two modelling problems considered.

Moreover, we have also presented estimates of the variability (uncertainty) in estimating the contributions to affinity, between various combinations of ligands and receptors, from different transmembrane regions. In Figure 5 (top), histograms display how often different kinds of descriptors were selected in the 500 models designed for the amine data set. One conclusions is that for corrfilter, the absolute valued cross terms are selected three times as often as ordinary cross terms. Another conclusion is that for PLSfilter, fewer variables are selected and there is no obvious preference for one of the two types of cross terms. For the alpha data set it is obvious from Figure 5 C that only TM2 and TM5 are important to the model. From Figure 6 C and 6D, it is also obvious that the cross terms (and also the absolute valued cross terms) are selected less often than the ordinary descriptors.

Figure 6 A and 6B displays contributions to affinity decomposed separately for each TM region and each drug/receptor combination. One conclusion here is that there is substantial variance in the estimates of the contributions which now is revealed and should dampen the risk of over-interpretations. Another conclusion is that the different regression and variable selection methods employed give similar results. Therefore, only one result each for the amine and the alpha data sets are presented in Figure 6. A third conclusion is that a more clear and more reliable pattern of contributions can be identified in the present study than from the estimated contributions in [2] which were based on a single model only. For example, a pattern of consistently negative average contribution is found from TM3 and the receptors 5HT1B to 5HT1F, but this pattern does not appear in Fig. 3 of [2]. A fourth conclusion is that for the alpha data set, there seem to be no significant contributions to affinity from TM1, TM3, TM4, TM6 and TM7. This result agree with previous results for this data set [2].

Although earlier findings have been confirmed, one should note that there are a number of differences between the present and earlier studies which makes detailed comparisons difficult: 1) In earlier work different variable subset selection methods were employed and in some attempts there were no subset selection at all. 2) The normalization and use of nonlinear cross terms differ between the present and earlier studies of the alpha data set. 3) The limited forms of external predictions attempted earlier e.g., in [2] are not directly comparable with the present results. 4) Different software packages have been employed for model selection and performance estimation.

## Conclusion

This work employs a methodology for unbiased statistical evaluation of proteochemometric modelling and confirms that proteochemometric modelling is a new bioinformatic methodology of great potential. The statistical evaluation performed on two of the largest proteochemometric data sets yet reported indicates that detailed chemical analyses of single proteochemometric models may be unreliable and that a systematic analysis of the set of different proteochemometric models produced in the statistical evaluation should yield more reliable information. Finally, although this work has focused on confirming the potential of proteochemometrics, the kind of systematic unbiased performance estimation employed here is of course also relevant for closely related areas of bioinformatics like microarray gene expression analysis and protein classification.

## Methods

### Data sets

In the amine data set, each of the 23 compounds was described by means of 236 different GRid INdependent Descriptors (GRIND) [26] computed for the lowest energy conformation found and organized into 6 different blocks associated with different kinds of physical interactions. In addition, each receptor was split into seven separate transmembrane regions by means of an alignment procedure and then each amino acid was described by means of five physico-chemical descriptors (z-scales). In total, 159 trans-membrane amino acids were translated into 795 physico-chemical descriptors organized into 7 different blocks (regions). In the alpha data set each of the 12 different compounds was described by means of 24 binary descriptors indicating the presence of different functional groups at three positions in the compound. Moreover, 52 amino acids in the trans-membrane regions of the receptors were identified to have varying properties between receptors and each of them were also coded into five or two physico-chemical properties each, yielding totally 96 descriptor values.

Before the proteochemometric modelling step, the amine data set was subjected to preprocessing in order to reduce the dimensionality of the original descriptors. This step should be part of the design procedure, leaving external examples outside. However, this issue is not expected to be critical and was therefore ignored in this study. For the compounds in the amine data set, after mean centering (no normalization), principal component analysis (PCA) was employed separately to each of six different blocks of GRIND descriptors, each block representing a particular kind of physical interaction. Similarly, each of the seven trans-membrane receptor block descriptors was subjected to PCA. This resulted in 6 × 10 = 60 compound descriptors and 7 × 15 = 105 receptor descriptors. Finally, 12,600 additional "cross-term" descriptors were produced by combining the compound and receptor descriptors nonlinearly. The cross-terms were added to account for non-linearities and they are shown to significantly improve the model predictivity. For each pair of compound and receptor descriptor blocks (totally 6 × 7 = 42 pairs), the 150 possible products between a compound and receptor descriptor value were computed. In addition, the absolute value of the deviation of each product from the average of the product over the data set available was computed. This resulted in 300 descriptor values for each of the 42 block pairs i.e., 42 × 300 = 12,600 values. For the alpha data set, the cross terms formed were the 2 × 24 × 96 = 4,608 possible products between the descriptors of ligands and receptors. No block-wise PCA was employed to reduce the dimensionality.

As a final step before entering the modelling phase, all descriptor values were mean centered and normalized to have unit variance.

### Robust PLS and ridge regression

In PLS regression, first a latent variable model

**x **= *t*_1_**b**_1 _+ *t*_2_**b**_2 _+ ... + *t*_*M*_**b**_*M *_    (2)

of the vector **x **of descriptor values is created where *t*_*m *_is latent variable and **b**_*m *_is the corresponding basis (loading) vector. As few uncorrelated latent variables as possible which have the largest covariances with the response variable *y*, are selected. Then, a linear model *y *= *a*_0 _+ *a*_1_*t*_1 _+ ... + *a*_*M*_*t*_*M *_is obtained from ordinary least squares fitting. Usually, this predictor is transformed back into the original variables yielding *y *= **w**^*T *^**x **as in (1). The robustness of PLS comes from the latent variable modelling which eliminates problems caused by strongly correlated variables and few examples. Ridge regression achieves its robustness by adding a penalty term (or, equivalently, a Bayesian prior) to the ordinary least squares criterion that reduces the variances in the regression coefficients. In the experiments considered below, the degree of penalty used in the RR and the number of latent variables used in the PLS regression were tuned in the inner CV loop to maximize their corresponding inner *K*_2_-fold cross validation performance estimates.

### Variable ranking algorithms

In the PLS modelling, the subsets of molecular descriptors used were selected jointly with the latent variables. Before the joint selection was performed, the molecular descriptors were ranked using two simple and fast methods: A bottom-up algorithm, which we call corrfilter, and a top-down algorithm which we call PLSfilter, corrfilter ranks the molecular descriptors according to the Pearson correlation coefficient between the descriptor and the response variable (the affinity). PLSfilter first builds a PLS model using all available descriptors and between one and *L *latent variables, where *L *is the number of latent variables associated with the model in (2) that explain 99% of the observed variance in *y*. Then each descriptor is ranked according to the corresponding mean of the squared coefficients, *w*_*i*_, in the regression models (1) from the *L *different models. For the alpha data set below only PLSfilter is applicable. This is due to the discrete nature of the ligand descriptors.

### Inner loop: joint VSS and regression parameter selection

After completing the variable ranking, the most promising combination of the number of top-ranked variables and the number of latent variables in the PLS regression modelling or the degree of penalty in the ridge regression modelling was selected as judged by a *K*_2_-fold CV performance estimate. The performance estimates for different combinations of values of *N*_*D*_, the number of top-ranked molecular descriptors, and values of *N*_*L*_, the number of latent variables (PLS) or degree of penalty (RR), were considered. Finally, the pair (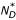, 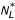) of numbers yielding the highest estimated predictive power was selected.

The predictive power of the models was measured by the commonly used dimensionless quantity *Q*^2 ^defined as


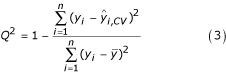


where *n *is the number of examples, *y*_*i *_is the measured biological activity of example *i*, 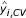 is the corresponding prediction, and 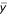 is the arithmetic mean value of all the measured activities. Hence, *Q*^2 ^is a CV estimate of the fraction of the variance of the response variable explained by the model. In the case of ordinary least squares fitting, *Q*^2 ^is also a CV estimate of the squared Pearson correlation coefficient between the true (*y*) and the predicted (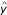) response values. Thus, a value of *Q*^2 ^close to one is traditionally interpreted as a good (valid) model.

### Outer loop: external *K*_1_-fold CV

As already mentioned, selection of a QSAR model that maximizes a *K*_2_-fold CV performance estimate is common in conventional chemometrics and is also applied in proteochemometrics. This method of tuning is more complicated and therefore slower than simpler alternatives (such as tuning to maximize a single conventional hold out performance estimate) but is expected to be less sensitive to overfitting. Although parameter tuning based on CV is attractive, overfitting may still occur and the performance estimate obtained may be too optimistic. Some aspects of this danger were recently pointed out [14] and has also been discussed in much earlier work [15]. In conclusion, it is important to employ a second external CV as in Figure 1 to estimate the true performance also of sophisticated design procedures that employ CV for parameter tuning.

For each step in the external *K*_1_-fold CV loop, one of the *K*_1 _subsets of the whole data set was kept for validation and the rest were used for design of a regression model. The predictions obtained in this outer CV loop were finally used in the formula for *Q*^2 ^in (3). However, since the predictions used for calculating *Q*^2 ^were kept outside the whole design procedure, as in earlier work [9,16], we denote the computed quantity by *P*^2 ^to indicate that this is an unbiased performance estimate based on external predictions.

### Repeated *K*_1_-fold CVs

The results obtained from a single *K*_1_-fold CV are interesting but are sometimes heavily influenced by the particular data partitioning used. In the work reported here, we therefore performed repeated *K*_1_-fold CV in the outer loop. For each partitioning selected randomly, the corresponding value of *P*^2 ^was computed using the procedures described above. Thus, a set of different values of *P*^2 ^were obtained and used for determination of the variability in the results obtained.

### Computations

The main body of programming and computations were performed using MATLAB on standard processors (900 MHz). For comparisons, we also employed the program packages SIMCA (Umetrics, Sweden), GOLPE [17] and UNSCRAMBLER (CAMO, Norway).

## Authors' contributions

E.F. and M.G. devised and implemented the proposed double CV loop procedure, the feature selection algorithms, and a numerically efficient version of ridge regression required. P.P., M.L., J.E.S.W. provided the data sets studied together with experience and insights gained from their earlier work on proteochemometrics. V.M. and M.G. supervised the project. All authors read and approved the final manuscript.

## Figures and Tables

**Table 1:** *Q*^2 ^and *P*^2 ^values for amine data set. The mean and standard deviations for the *P*^2 ^and *Q*^2 ^values obtained with the amine data set using two different variable selection methods (corrfilter and PLSfilter) and two different regression methods (PLS and RR). The 5-fold cross validation procedure was repeated 100 times, using 100 different random partitions of the data. *N*_*D *_and *N*_*L *_values were selected in an inner 5-fold cross validation loop by optimizing the *Q*^2 ^value. For one random partition of the amine data into five cross validation groups, one *P*^2 ^and five *Q*^2 ^values were obtained. For every random partition the mean *Q*^2 ^is computed. The mean and standard deviations were computed based on the 100 *P*^2 ^values and the 100 mean *Q*^2 ^values.

Filter	Regression	*P*^2 ^(mean ± std)	mean *Q*^2 ^(mean ± std)
no filter	PLS	0.52 ± 0.021	0.49 ± 0.011
no filter	RR	0.53 ± 0.022	0.49 ± 0.012
corrfilter	PLS	0.49 ± 0.028	0.76 ± 0.0057
corrfilter	RR	0.44 ± 0.038	0.76 ± 0.0085
PLSfilter	PLS	0.52 ± 0.025	0.90 ± 0.0026
PLSfilter	RR	0.51 ± 0.027	0.90 ± 0.0056

**Table 2:** *N*_*L *_and *N*_*D *_for amine data set. The mean and standard deviation of the number of latent variables or degree of RR penalty (*N*_*L*_) and the number of molecular descriptors (*N*_*D*_) used to build the models for the the amine data set. The values of *N*_*L *_and *N*_*D *_are tuned by optimizing the *Q*^2 ^value in an inner cross validation loop.

Filter	Regression	*N*_*L *_(mean ± std)	*N*_*D *_(mean ± std)
no filter	PLS	6.49 ± 1.48	12765 ± 0
no filter	RR	1.13 ± 1.45	12765 ± 0
corrfilter	PLS	7.00 ± 0.88	4748 ± 730
corrfilter	RR	1.83 ± 1.18	4871 ± 640
PLSfilter	PLS	6.18 ± 1.40	1933 ± 455
PLSfilter	RR	2.10 ± 1.17	2136 ± 349

**Table 3:** *Q*^2 ^and *P*^2 ^values for alpha data. set The mean and standard deviations for the values of *Q*^2 ^and *P*^2 ^obtained for the alpha data set using the variable selection method PLSfilter and the regression methods PLS and RR.

Filter	Regression	*P*^2 ^(mean ± std)	mean *Q*^2 ^(mean ± std)
no filter	PLS	0.55 ± 0.045	0.50 ± 0.066
no filter	RR	0.65 ± 0.037	0.59 ± 0.027
PLSfilter	PLS	0.77 ± 0.033	0.83 ± 0.0095
PLSfilter	RR	0.76 ± 0.043	0.83 ± 0.010

**Table 4:** *N*_*L *_and *N*_*D *_for alpha data set. The mean and standard deviation of the number of latent variables or degree of penalty (*N*_*L*_) and the number of molecular descriptors (*N*_*D*_) used to build the models for the the alpha data set.

Filter	Regression	*N*_*L *_(mean ± std)	*N*_*D *_(mean ± std)
no filter	PLS	7.01 ± 1.15	4728 ± 0
no filter	RR	93.64 ± 60.34	4728 ± 0
PLSfilter	PLS	7.39 ± 0.80	199 ± 69
PLSfilter	RR	26 ± 22	192 ± 85
